# Does medical school research productivity predict a resident’s research productivity during residency?

**DOI:** 10.1186/s40463-017-0202-6

**Published:** 2017-04-27

**Authors:** Scott Kohlert, Laura Zuccaro, Laurie McLean, Kristian Macdonald

**Affiliations:** 1 Department of Otolaryngology, The Ottawa Hospital, 501 Smyth Rd, Ottawa, ON K1H 8L6 Canada; 2 University of Ottawa, Ottawa, ON Canada

**Keywords:** Residency, CanMEDS, Publishing, Resident selection, Otolaryngology

## Abstract

**Background:**

Research productivity is an important component of the CanMEDS Scholar role and is an accreditation requirement of Canadian Otolaryngology training programs. Our objective was to determine if an association exists between publication rates before and during Otolaryngology residency.

**Methods:**

We obtained the names for all certified Canadian Otolaryngologists who graduated between 1998 and 2013 inclusive, and conducted a Medline search for all of their publications. Otolaryngologists were subgrouped based on year of residency graduation and the number of articles published pre-residency and during residency (0 or ≥1). Chi-squared analyses were used to evaluate whether publications pre-residency and year of graduation were associated with publications during residency.

**Results:**

We obtained data for 312 Canadian Otolaryngologists. Of those 312 graduates, 46 (14.7%) had no identifiable publications on PubMed and were excluded from the final data analysis. Otolaryngology residents had a mean 0.65 (95% CI 0.50-0.80) publications before residency and 3.35 (95% CI 2.90-3.80) publications during residency. Between 1998 and 2013, mean publication rates before and during residency both increased significantly (*R* ^2^ = 0.594 and *R* ^2^ = 0.759, respectively), whereas publication rates after residency graduation has stagnated (*R* ^2^ = 0.023). The odds of publishing during residency was 5.85 times higher (95% CI 2.69-12.71) if a resident published prior to residency (*p* < 0.0001). The Spearman correlation coefficient between publications before and during residency is 0.472 (*p* < 0.0001).

**Conclusion:**

Residents who publish at least one paper before residency are nearly six times as likely to publish during residency than those who did not publish before residency. These findings may help guide Otolaryngology program selection committees in ranking the best CaRMS candidates.

## Background

Medical students compete each year for approximately 30 Otolaryngology - Head and Neck Surgery (OTOHNS) residency positions country-wide through the Canadian Residency Matching Service (CaRMS). In 2015, 60 Canadian medical graduates applied for the 29 available residency positions, making OTOHNS the third most competitive surgical discipline [1]. Candidates are ranked based on their academic record, letters of recommendations, personal statements, elective experience, publications and research experience, extracurricular talents, personality and interpersonal traits, and overall impression. These factors, combined with their performance during face-to-face interviews, are used to assign an overall final rank for the match process.

There is previous literature that can help program selection committees predict which applicants will be most successful during residency [2, 3]. In 2012, Chole and colleagues found that many of the application factors typically used to select Otolaryngology resident candidates—such as exam performance, letters of recommendation, and performance during internship—might not be predictive of future capabilities as a clinician. In addition, Alpha Omega Alpha membership and United States Medical Licensing Examination (USMLE) Part 1 examinations were not correlated with physician success [4]. However, rank of the medical school and faculty interview, as well as having excelled in a team sport correlated with higher clinical performance [5]. Previous studies that attempted to demonstrate an association between clinical performance in medical school and residencies in other surgical subspecialties have shown mixed results [6, 7].

Most medical schools and residency programs now support and expect completion of a scholarly project as part of postgraduate training. A recent study by Chen and colleagues showed a significant increase in the number of resident publications in the last few years [8]. While it is generally felt that publishing as a medical student and/or resident helps demonstrate proficiency in the CanMEDS “Scholar” role [9], a study involving general internal medicine residents [10] revealed that having pre-residency publications was not associated with higher evaluations in the scholar category. That said, previous work has demonstrated that publishing as a medical student or resident is significantly associated with subsequent career publications [11–13] and that medical school publications are associated with a higher propensity towards an academic career after completion of residency [14]. Of note, all data in these prior studies comes from medical and surgical specialties other than Otolaryngology.

We theorized that research productivity in medical school was associated with research productivity in OTOHNS residency. A recent study by Gupta and colleagues found that publishing prior to residency is significantly associated with publishing during a Pediatrics residency [15]. While prior research may be valued during the CaRMS selection process for applicants to an OTOHNS residency program, to our knowledge no similar study has examined this link among applicants to competitive surgical specialties. Our objective was to identify whether an association exists between publishing before and during OTOHNS residency. Our secondary outcome was to evaluate the trend of research productivity among medical students, residents and attending physicians over time.

## Methods

### Participants

This study targeted all practicing otolaryngologists who were certified by the Royal College of Physicians and Surgeons of Canada (RCPSC) between 1998 and 2013, inclusive. We created a database from information contained in the publicly available Royal College Specialist Directory [16] which included each surgeon’s last name, first name, middle names, certification date and current city of practice.

### Publication collection

Each otolaryngologist was searched by full name in PubMED and any publications matching to that author were recorded. To increase the sensitivity of our search, multiple permutations were used for each search query (Table 1). In an attempt to reduce the likelihood of false positive identification, we used the following supportive characteristics for each publication collected: publication topic of Otolaryngology, corresponding geographic location of affiliated institution, and matching middle initials between publication and RCPSC database. If multiple authors with the same name were found, publications were only attributed to the target otolaryngologist if at least one of the previously mentioned supporting characteristics was present. To further increase the accuracy of our search, publication lists were cross-referenced with any identifiable publically available external sources including Research Gate, LinkedIn and online curriculum vitaes (CVs). Missed publications were added and false positives were removed from the final list of publications. Data collection was performed on April 1, 2015, and no papers published after this date were captured in this study.

**Table 1:** Search permutations used to collect publications from PubMed

Search Permutations
Last, First
Last, First Middle
Last, F
Last, F*
Last, FM

*PubMed truncation symbol

### Publication categorization

**Fig. 1:**
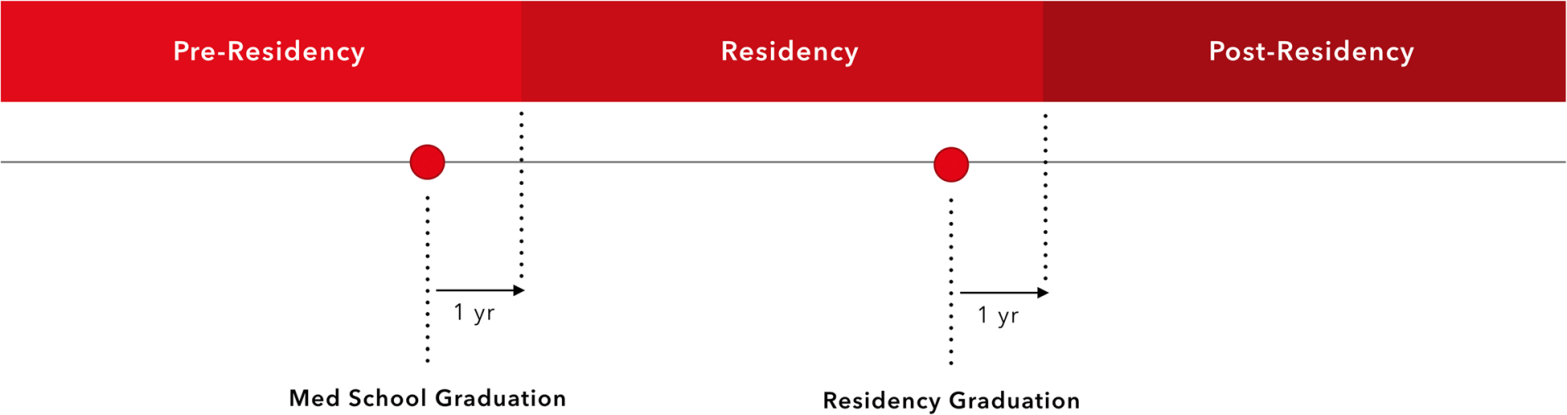
Timeline used to designate pre and during residency publication periods

Otolaryngology – Head and Neck Surgery (OTOHNS) certification in Canada is completed in the summer of each year on June 30, following the final RCPSC examinations. The expected during residency and pre-residency time periods were classified based on the certification date. OTOHNS residency in Canada is five years in duration, thus the five years preceding certification were designated as residency, and prior to this was considered pre-residency. Each period was extended by one year, to capture articles that were likely completed within the pre or during residency period, but not published until afterwards (see Fig. 1). Articles were then categorized based on their date of initial abstract submission.

### Statistical analysis

Each otolaryngologist was categorized as having either 0, 1, 2 or ≥ 3 publications for both of the time periods. Descriptive statistics were performed, including the mean rates of publication in each group, with 95% confidence intervals (95% CI). The data was separated by certification year in order to calculate trends in average publication rates over time using linear regression (Coefficient of Determination, *R* ^2^). Chi-squared analyses were used to evaluate whether publications pre-residency and year of graduation were associated with publications during residency. A Mantel-Haenszel calculation was used to measure the combined odds-ratio for the study population. A Spearman correlation coefficient was calculated for the relationship between the number of publications before residency compared to the number during residency.

### Validation

To validate whether this method truly identifies a clinician’s list of publications, an email was sent to all members of the Canadian Society of Otolaryngology who graduated between the years of 1998 and 2013 asking for a copy of their CV, as it was assumed that the CV could be used as the gold standard list of an author’s publications (relying on the assumption that the authors would maintain an accurate list of their publications). This CV was subsequently cross-referenced against the list of publications that had been identified in our search. Any publications meeting the following exclusion criteria outlined in Table 2 were removed from the list.

**Table 2:** Validation Process - Exclusion Criteria

1. Papers published after April 1, 2015 (the date of data collection)
2. Articles published in non peer-reviewed journals, or in biomedical journals that are not indexed by Pubmed (as this is a basic marker of journal quality) including popular magazines and newspaper articles
3. Textbook chapters
4. Patents

Papers meeting the inclusion criteria were then manually cross-referenced against the publications identified in our search. True positives, false positives and false negatives were tracked and used to determine sensitivity and positive predictive value.

### Ethics and permissions

As all data was publically available on PubMed and the Royal College website, research ethics board approval was not necessary for this study.

## Results

### Publication rates

Using the Royal College database and Medline/PubMed, 3441 publications for 312 residents were identified between 1998 and 2013. 46 (14.7%) of these had no publications during their career. There was an average of 0.65 (95% CI 0.50-0.80) publications before residency and 3.35 (95% CI 2.90-3.80) publications during residency. The number of residents with no publications was 216 (63%) prior to residency compared to 83 (26.6%) during residency. Only 7% (23/312) had ≥3 publications before residency, while 42% (131/312) had ≥3 publications during residency.

Residents who had at least 1 publication prior to residency were nearly six times more likely to publish at least once during residency (OR 5.85; 95% CI 2.7-12.7; *p* < 0.0001). There was a linear correlation between research publications prior to and during residency (*r* = 0.472, *p* < 0.0001). Table 3 shows the mean, median and mode publication rates among residents before and during residency.

**Table 3:** Overall mean, median and mode publication rates among Otolaryngology residents from 1998-2013

Number of publications	Before residency	During Residency
Mean	0.65	3.35
Median	0	2
Mode	0 (216/312 residents)	0 (83/312 residents)

### Overall publication trends

**Fig. 2:**
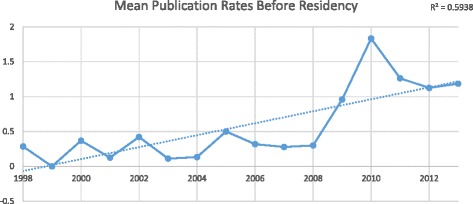
Publications per resident *before* residency by graduation year

**Fig. 3:**
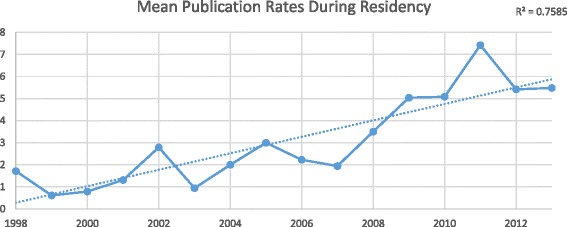
Publications per resident *during* residency by graduation year

**Fig. 4:**
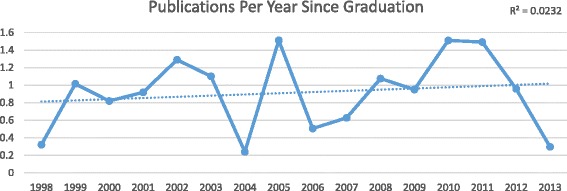
Publications per resident *after* residency by graduation year

Between 1998 and 2013, publication rates before and during residency both increased significantly, whereas publication rates after residency graduation stagnated. Residents who graduated from residency in 1998 had an average of 0.3 publications prior to residency, compared to 1.2 publications for graduates from 2013 (*R* ^2^ = 0.594). Individuals completing residency in 1998 published an average of 1.7 publications during residency, compared to 5.5 publications for those finishing in 2013 (*R* ^2^ = 0.758). This strong trend of increasing research productivity over time did not persist when looking at publication rates after completion of residency. Following graduation from Otolaryngology residency, individuals published an average 0.6 publications/year in 1998 compared to 0.3 publications/year in 2013 (*R* ^2^ = 0.023). Figures 2, 3, 4, and Table 4 highlight the trends in mean and median publication rates over time.

**Table 4:** Mean and median publication rates by residency graduation year

	Before Residency *(total number of publications)*		During Residency *(total number of publications)*		After Residency *(number of publications per year)*	
*Grad Year*	Mean	Median	Mean	Median	Mean	Median
1998	0.29	0	1.71	0	0.32	0.00
1999	0.00	0	0.62	0	1.02	0.33
2000	0.37	0	0.79	0	0.82	0.07
2001	0.13	0	1.31	0	0.92	0.31
2002	0.42	0	2.79	1	1.29	0.17
2003	0.11	0	0.94	1	1.10	0.09
2004	0.13	0	2.00	2	0.24	0.10
2005	0.50	0	3.00	2	1.51	0.28
2006	0.32	0	2.23	2	0.51	0.13
2007	0.28	0	1.94	2	0.63	0.36
2008	0.30	0	3.50	2	1.08	0.33
2009	0.96	0	5.04	4	0.95	0.40
2010	1.83	1	5.08	4	1.51	0.75
2011	1.26	0	7.42	5	1.49	0.67
2012	1.13	1	5.42	4	0.96	0.50
2013	1.19	1	5.48	4	0.30	0.00

### Validation

Thirty-one authors (10.3% response rate) provided us with either a copy of their CV or a separate up-to-date list of their publications. Each of the 874 publications that met the inclusion criteria were cross-referenced against the list of publications identified in our initial study. We identified 35 missed publications (i.e. false negatives), resulting in a search sensitivity of 96.1%. Based on the validation data, we identified an average of 1.1 missed publications per author.

The validation process initially identified 15 articles (from a total of 5 authors) as false positives as they were not found on the CV provided by the authors. These 5 authors were contacted to verify the accuracy of their CV. Upon further investigation, each of these 15 publications were in fact accurately attributed to the author in question (leaving us with zero false positives, and a positive predictive value of 100%).

## Discussion

Compared to residents who did not publish prior to residency, we found that Otolaryngology residents who published at least once prior to residency were nearly six times as likely to publish in postgraduate training. Our results suggest a moderately correlated linear relationship of the number of papers published before residency with the number during residency. These findings indicate that research conducted prior to residency increases the likelihood the resident will publish scholarly work after residency begins.

Despite the demonstrated association between pre-residency publication and publications during residency, other variables are likely also at play and further work is required to identify and account for these confounding factors. Not all residency candidates have research experience, and publishing as a medical student or resident has not been shown to make an individual a better clinician. As previously stated, nearly 15% of Otolaryngologists in our study did not have any identifiable publications. Further, pre-residency publication status is not a definite predictor of a resident’s publication potential, as 65% (141/216) of individuals without pre-residency publications did proceed to publish as a resident.

The results from this study could be used to help guide the research curriculum within Otolaryngology residency programs. For example, residents without prior research experience may benefit from early mentorship and formal training in research skills, and residents with prior experience may benefit from an amount of protected research time proportional to their research interests.

### Potential limitations

Although a strength of our dataset is that it is a recent comprehensive national population study not affected by response rates, the correct author and publication time period could not be confirmed on an individual basis. Individuals whose residency periods were extended to greater than five years (e.g. due to completion of masters’ degrees, maternity leave, or examination failure) may not be accurately identified in our analysis. This could result in misclassification of publications into the wrong time period (pre vs. during residency) and could skew our results either towards or away from the null hypothesis (that there is no correlation between pre or during residency publications).

Further, PubMed was exclusively used for data collection and any publications not listed on PubMed were not captured in the dataset, potentially underestimating the true number of a researcher’s publications. We exclusively used this search method because of the basic scientific rigor associated with the PubMed’s abstract listing criteria [17].

### Future directions

Future research may include attempting to further stratify candidates based on various personal, program and publication variables.

Personal variables include: an individual’s previous graduate degrees held, medical school, and completion of a fellowship. Another interesting personal variable is the individual’s *h-index,* an objective and easily calculable measure to evaluate both the number, as well as the relative importance of an author’s scientific contributions. By looking not only at the number of publications but also the number of times each paper has been cited in the literature, the *h-index* is considered to be a more accurate marker of publication quality and academic success [18].

Future studies could also investigate the effect that the research environment (residency program, dedicated research time, support resources, attending physician research productivity, etc) has on the resident’s research productivity.

Publication variables that could be examined include type of publication (eg: case report vs. systematic review vs. randomized control trial) and journal impact factor. Furthermore, future studies could also investigate the authorship trends of research in Otolaryngology. Single authorship is becoming increasingly less common in our current academic world, and inappropriate assignment of authorship credit is an increasingly well known phenomenon [19]. For example, a survey of authors in the “basic and medical sciences category” found that 26% of non-first authors admitted to not contributing substantially to the paper [20]. Otolaryngology may be not immune to this phenomenon, with multiple authors in our study having been credited with publishing at an average rate of ≥1 paper/month over the past 5 years. Further studies looking at research productivity could evaluate author order, degree of author contribution, and total number of co-authors listed per publication.

While our study found that publication rates after residency graduation have not been rising over time, our data was not detailed enough to allow us to separate academic otolaryngologists from community otolaryngologists. A future study could collect this data and perform a subgroup analysis to compare the trends in research productivity over time between these two groups.

## Conclusion

In this nation-wide sample of Canadian certified otolaryngologists, we demonstrated that pre-residency publication is significantly associated with subsequent publication during residency. Over the past 16 years, publication rates have steadily increased both before and during residency. During that same timespan, publication rates among practicing otolaryngologists has remained relatively stagnant. Our analysis did not take into account several potential confounding variables.

Pre-residency research productivity is a predictor for research productivity in residency and can act as a helpful marker for residency program selection committees in ranking candidates for the CaRMS match.

## Abbreviations

OTOHNS: Otolaryngology - Head and Neck Surgery
CaRMS: Canadian Residency Matching Service
USMLE: United States Medical Licensing Examination
RCPSC: Royal College of Physicians and Surgeons of Canada
CV: Curriculum vitae
*R*^2^: Coefficient of Determination
